# Automatic classification of patients with myocardial infarction or myocarditis based only on clinical data: A quick response

**DOI:** 10.1371/journal.pone.0285165

**Published:** 2023-05-05

**Authors:** Sheikh Shah Mohammad Motiur Rahman, Zhihao Chen, Alain Lalande, Thomas Decourselle, Alexandre Cochet, Thibaut Pommier, Yves Cottin, Michel Salomon, Raphaël Couturier

**Affiliations:** 1 FEMTO-ST Institute, CNRS UMR 6174, Univ. Bourgogne Franche-Comté, Belfort, France; 2 ICMUB Laboratory, CNRS UMR 6302, Faculté de Médecine, Univ. Bourgogne Franche-Comté, Dijon, France; 3 Dijon Bourgogne University Hospital, Dijon, France; 4 Société CASIS – CArdiac Simulation & Imaging Software, Dijon, France; Sejong University, KOREA, REPUBLIC OF

## Abstract

**Background:**

In acute cardiovascular disease management, the delay between the admission in a hospital emergency department and the assessment of the disease from a Delayed Enhancement cardiac MRI (DE-MRI) scan is one of the barriers for an immediate management of patients with suspected myocardial infarction or myocarditis.

**Objectives:**

This work targets patients who arrive at the hospital with chest pain and are suspected of having a myocardial infarction or a myocarditis. The main objective is to classify these patients based solely on clinical data in order to provide an early accurate diagnosis.

**Methods:**

Machine learning (ML) and ensemble approaches have been used to construct a framework to automatically classify the patients according to their clinical conditions. 10-fold cross-validation is used during the model’s training to avoid overfitting. Approaches such as Stratified, Over-sampling, Under-sampling, NearMiss, and SMOTE were tested in order to address the imbalance of the data (i.e. proportion of cases per pathology). The ground truth is provided by a DE-MRI exam (normal exam, myocarditis or myocardial infarction).

**Results:**

The stacked generalization technique with Over-sampling seems to be the best one providing more than 97% of accuracy corresponding to 11 wrong classifications among 537 cases. Generally speaking, ensemble classifiers such as Stacking provided the best prediction. The five most important features are troponin, age, tobacco, sex and FEVG calculated from echocardiography.

**Conclusion:**

Our study provides a reliable approach to classify the patients in emergency department between myocarditis, myocardial infarction or other patient condition from only clinical information, considering DE-MRI as ground-truth. Among the different machine learning and ensemble techniques tested, the stacked generalization technique is the best one providing an accuracy of 97.4%. This automatic classification could provide a quick answer before imaging exam such as cardiovascular MRI depending on the patient’s condition.

## Introduction

One of the most frequent cardiovascular diseases is myocardial infarction [1]. Myocarditis [2], also known as inflammatory cardiomyopathy, on the other hand, is a relatively difficult condition to diagnose easily. Clinical information and Delayed Enhancement Magnetic Resonance Imaging (DE-MRI, imaging several minutes after the injection of a contrast agent) are suitable for the diagnosis of myocardial infarction and myocarditis. Globally, myocardial infarction is a leading cause of mortality and disability. Coronary artery disease is a long-term condition with phases of stability and instability. Patients may experience a myocardial infarction during unstable duration with active inflammation in the myocardial wall in the acute phase. Myocardial infarction can be a modest complication of a long-term chronic condition that goes unnoticed, or it can be a big catastrophe that results in a sudden death or major haemodynamic deterioration, particularly during an acute phase. A myocardial infarction can happen repeatedly [3].

Acute heart failure [4], abrupt death, and persistent dilated cardiomyopathy can be caused by myocarditis years later, if the pathology was under-diagnosed. Because myocarditis has non-specific symptoms including chest discomfort, dyspnea, and palpitations, it might be mistaken for more serious conditions such as coronary artery disease [5].

If myocardial infarction or myocarditis is suspected, the patient undergoes medical imaging exams such as MRI [6]. Indeed, cardiac MRI (CMR) [7] is a powerful diagnostic method, allowing robust measurements of crucial markers of cardiac structure and function. In particular DE-MRI displays the diseased heart as hyperenhanced area, both in myocardial infarction and in myocarditis. The main differences are the shape and the localization of the abnormal areas.

However, a limitation in the management of the patient is that there is often a delay between the admission of the patients in the emergency department and the CMR exam. In this context, early diagnosis would be ideal. Therefore, early diagnosis from clinical data can be an instant solution for a rapid decision for initial care. However, patients who have had a myocardial infarction or myocarditis have much in common in terms of clinical behavior. Therefore, it can be difficult to distinguish between these two cardiac disorders without the results of the MRI exam. Thus, this study targeted patients with suspected myocardial infarction or myocarditis to initially classify these types of patient with other clinical conditions that get a normal DE-MRI exam.

We propose to use the patients’ clinical data recorded during their admission in the emergency department to anticipate the conclusion of the imaging exam. The main issues are to categorize cardiac disorders using solely clinical data. A previous work [8] introduced a method allowing the distinction between myocardial infarction and normal individuals. This previous study did not consider cases with myocarditis. Then, myocarditis cases are included in this study. Secondly, an optimized solution, applicable for real scenarios that have imbalance issues, in practice, was designed. To sum up, we provide a method that not only categorizes the diseases based only on clinical data, such as patient with normal DE-MRI exam, myocarditis or myocardial infarction, but also fixes the data imbalance issues that are encountered in this kind of applications. Additionally, a wrapper method has been employed to reduce the feature set used to classify the patients and compute the weight of each features based on its importance for a particular model training.

More specifically, issues of overfitting and underfitting [9] are taken into consideration, both of which are common problems in machine learning based solutions. Mostly, the trends of overfitting or underfitting are due to the nature of data samples in practice (neither balanced nor large enough). Thus, the proposed model used 10-fold cross-validation along with the stratified splitting technique (maintaining the same ratio of each class in train and test) to tackle the mentioned problems. In addition, another 10-fold cross-validation has been implemented within the stacked generalization technique (Stacking) [10] which is an ensemble machine learning based multi-level approach. Stacking is used to minimize the error bias in training so that maximum optimized performance can be reached.

The major objective of this work is to classify the patients according to the clinical data, with the result of DE-MRI as reference. More specifically, the focus of this research work is to achieve automatic classification of patients into one of three classes based on their clinical data: patients with myocardial infarction, patients with myocarditis or patients with another clinical condition and normal DE-MRI exam.

The main contributions of this study are:

- Automatic classification tool of patients with cardiovascular diseases especially myocardial infarction and myocarditis.
- Assessment and evaluation of traditional machine learning classifiers, ensemble classifiers and Multi-Layer Perceptron (MLP) in the field of cardiovascular disease classification.
- Evaluation of the different combinations of classifiers and data unbalancing techniques to identify the best combination among them that can manage unbalanced data.

## Related works

Pellaton et al. [11] aimed to investigate the prevalence and diagnostic severity of myocardial infarction (MI) and myocarditis in young adults who were admitted to the emergency department (ED) with chest pain (CP) and an elevated serum troponin I (TnI). They considered 1,588 patients during 30 consecutive months, aged between 18 and 40 years old. In their study, 32.7% of people younger than 40 years old patients with elevated TnI were diagnosed with MI. Diabetes, dyslipidemia, a familial history of coronary artery disease (CAD), a fever or recent viral infection also are all important clinical features to consider. Thus, this shows the importance of clinical features in the classification of myocardial infarction (MI), myocarditis and normal patients.

For the classification of myocardial infarction from multi-lead ECG data, Chang et al. [12] used Gaussian mixture models (GMM) and hidden Markov models (HMM). In their study, GMM with varied numbers of distributions grouped the 4-dimension feature vector collected by hidden Markov models (disease and normal data). During this study, there were 1,129 samples of ECG, with 582 samples from patient with myocardial infarction and 547 samples from healthy people. Their methodology provided 85.71% of sensitivity, 79.82% of specificity and the accuracy was 82.50%.

Consecutive MRI exams of 111 patients with MI and 62 patients with myocarditis with DE-MRI were included in the work of Di Noto et al. [13]. Classification results from two-dimensional (2D) and three-dimensional (3D) texture analysis, shape, and first-order descriptors were compared using five different machine learning techniques. The authors used a nested, stratified 10-fold cross-validation method. The effects of resampling MR images were investigated using both supervised and unsupervised feature selection strategies. They claimed that DE-MRI’s radiomic characteristics allow for a high-accuracy differentiation between MI and myocarditis utilizing either 2D features and Recursive feature elimination (RFE) or 3D features with PCA.

Baloglu et al. [14] proposed multi-lead ECG signals and a deep convolutional neural network-based architecture for classification of different types of MI. During their assessment, they considered 10 types of MI extracted from the public physiobank ECG dataset. To detect myocardial infarction in signals of I-lead ECG, Feng et al. [15] have proposed an automatic multi-channel classification algorithm with a 16-layer convolutional neural network (CNN) along with long-short term memory network (LSTM). Their algorithm first extracted the heartbeat segments from the raw data before training the multi-channel CNN and LSTM to learn the acquired features. To validate their approach, they used the Physikalisch-Technische Bundesanstalt (PTB) database and obtained a 95.4% accuracy rate, a sensitivity of 98.2%, a specificity of 86.5%, and a F1-score of 96.8%, indicating that the model can achieve good classification performance without complex handcrafted features.

Shi et al. [16] presented a mixed classification algorithm that takes both clinical features and DE-MRI into account to efficiently learn the association between these variables and automatically predict if a patient has myocardial infarction. A 3D convolutional neural network (CNN) encodes the MRI as a surface of a diseased area in the mixed model, and the surface is then fed into Random Forest with other clinical characteristics to determine the final choice. Lourenço et al. [17] proposed a deep learning neural network based approach that can automatically predict myocardial disease based on patient clinical data and DE-MRI. All of the proposed networks have a high level of classification accuracy (greater than 85%). In this classification task, including information from DE-MRI (directly as images or as metadata following DE-MRI segmentation) is beneficial, increasing accuracy to 95-100% on the same dataset. Girum et al. [18] proposed a deep learning framework with cross-validation for the classification of the patients with or without myocardial infarction. Using five-fold cross-validation, the classification based solely on clinical data yielded an accuracy of 80% according to their statement. In addition, this technique can categorize patients with 93.3% of accuracy using also DE-MRI. These last three studies validated their framework on the EMIDEC MICCAI challenge dataset [19].

In fact, one study [20] proposed an ensemble deep learning based model to predict the existence of heart diseases or cardiovascular diseases which enable to handle the high-dimensional heart diseases data. They employed feature fusion, feature selection, weighting techniques and obtained 98.5% of accuracy in heart disease classification. However, this framework enables the basement idea of smart heart disease monitoring system based on deep learning techniques but not focused on any specific types of disease such as myocardial infarction and myocarditis.

It can be concluded that the classification of myocardial infarction, myocarditis and other diseases among patients is attracting great attention from researchers. Nevertheless, the works in the literature are primarily focused on MRI, not on clinical data alone. In fact, only one study proposed a solution to classify patients with myocardial infractions or not, but myocarditis is not considered. In this study, we propose a technique that not only classifies diseases based on clinical data, including myocarditis, but also resolves the imbalance problems that actually exist in practice.

In sum up from the literature, it is clearly understandable from the state-of-art that researchers are trying to find the best approach to quickly classify patients with pathological diseases. Most of the researchers worked on the automatic classification of MI types, some on the classification between MI and normal case, but considering also myocarditis is an important and emerging task today. Our study will provide a quick response in classification of myocardial infarction, myocarditis or patients with other condition and normal DE-MRI. In addition, machine learning techniques have been evaluated on a large scale with tackling overfitting and data imbalance issues that exists in real clinical practice.

## Dataset information

The data came from the dataset used in the EMIDEC [19] challenge in addition to exams of patients with myocarditis (all the exams were acquired in the University Hospital of Dijon (France) with the same protocol). Our study was conducted in accordance with the principles of good clinical practice and followed both the French legislation and the university ethical committee (certification from the French Committee for the Protection of Persons (CPP) unit Est 1). The need for informed consent was waived, but all participants were given clear information about the study, and their non-opposition was obtained. Additional information from patients with myocarditis were collected specifically for the purpose of this study. These information were separate from the EMIDEC challenge dataset.

The EMIDEC challenge dataset [19] consists of DE-MRI scans with associated clinical information from 150 patients. For one exam, there is a series of DE-MRI images in short axis orientation of the heart from the base to the apex of the left ventricle, along with ground truths (i.e. contours of the myocardium and diseased areas). DE-MRI examinations typically contain 7 slices for each case. Additionally, there is a text file which contains the clinical data. The distribution of normal (1/3) and pathological (2/3) instances is imbalanced, roughly reflecting real life in an MRI department. Patients admitted to a cardiac emergency department with signs of a heart attack falls into this study’s target group. The presence or absence of a disease area on DE-MRI was used to characterize each group. In our population, patients with myocardial infarction (MI) were both STEMI (ST-elevation myocardial infarction) or NSTEMI (Non-ST-Elevation Myocardial Infarction), in accordance with ECG initial presentation. Moreover, they could include coronary endothelial dysfunction and platelet activation leading to subsequent coronary thrombosis (type 1 MI), and also others conditions like sepsis or arrhythmia related increasing in myocardial oxygen consumption in the absence of atherothrombotic events (type 2 MI). Patients with multiple pathologies were rejected. Among clinical characteristics available in the EMIDEC dataset, 10 were retained for our study: sex, age, use of tobacco (Yes, No, or former smoker), overweight (if BMI is greater than 25), hypertension (Y/N), diabetes (Y/N), familial history of coronary artery disease (Y/N), troponin (value), ejection fraction of the left ventricle from echocardiography (value), and NT-proBNP (value). Indeed, two clinical characteristics present in the EMIDEC dataset were not retained because they are not appropriate for the myocarditis cases: it is the type of myocardial infarction from ECG (ST+ (STEMI) or not) and the Killip max. As shown in Table 1, the value of each feature can be categorical, Boolean, or float.

**Table 1:** The 10-features considered during this study.

Features Name	Description	Example
Sex	0 = Male, 1 = Female	0
Age	Age in years (Number)	55
Tobacco	1 = Yes, 0 = No, 2 = Former Smoker	2
Overweight	If BMI>25 then Yes else No	1
Hypertension	Yes or No	1
Diabetes	Yes or No	1
Familial Disease	Familial history of Cardiovascular Events (Yes or No)	0
Troponin	Value (ng per mL)	9.10
LEVF	Calculated from echocadiography (%)	62
NT-proBNP	Value (pg per mL)	351

In details, the patient’s entire past acute cardiac episodes were included in the familial history of coronary artery disease. A troponin test determines the amount of troponin T or troponin I proteins in the blood. These proteins enter the bloodstream when the heart muscle is damaged, such as during a myocardial infarction. In general, a result of 0.1 or less is thought to be normal, whereas a value of 0.4 or above is thought to be abnormal. The peptide NT-pro-brain natriuretic peptide (NT-proBNP) is a marker for heart failure diagnosis that is tested in venous blood [21]. Natriuretic peptides are hormones that have vasodilator properties and are mostly released in the left ventricle as a pressure-compensating mechanism. Normal is defined as a NT-proBNP value lower than 135 units. Tropinin and NT-proBNP cannot discriminate easily between MI and myocarditis at the acute phase. During the patient’s admission to the emergency room, the left ventricular ejection fraction (LVEF) is estimated using standard echocardiography.

According to the results of the DE-MRI, there were 50 patients presenting normal exams, 100 patients with myocardial infarction, and 179 patients with myocarditis.

## Methods

### Developed approach

**Fig 1:**
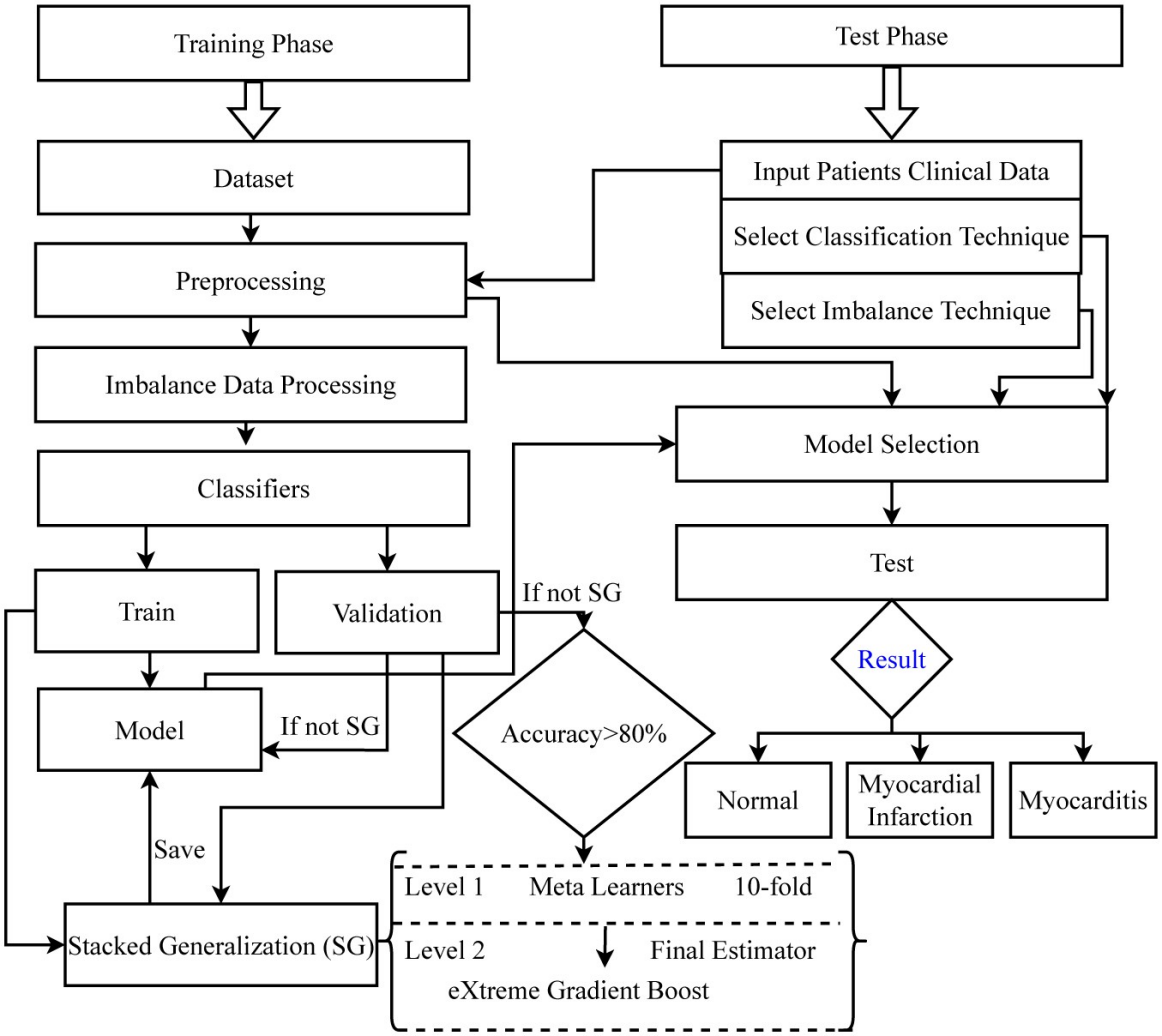
Architecture of the proposed approach.

In this section, the developed approach that has been depicted in Fig 1 is discussed in details. The training process begins with the preprocessing of the data, followed by the selection of an imbalance technique and finally a classification algorithm.

#### Preprocessing

In the preprocessing step, we have standardized the data format in a uniform way using the standard scalar technique. Thus, we have only numerical values, we used Normalize filter [22] which limits the values between 0 and 1. The standard score *Z* of a data sample *x* is calculated according to Eq (1).

$$\begin{array}{c} Z=\frac{x-\mu }{\sigma }, \end{array}$$ (1)

where

- *Z* = Standard score or z-score.
- *x* = A data sample (the values of features) in dataset.
- *μ* = Mean of the training samples.
- *σ* = Standard deviation of the training samples.

#### Imbalance data processing

Unbalancing techniques like Stratified [23, 24], Under-sampling [25], Over-sampling [26], NearMiss [25, 27] and Synthetic Minority Over-sampling Technique—SMOTE [28–30] have been implemented along with 10-fold cross-validation for tackling imbalance and overfitting issues. Cross-validation (CV) is a method used to improve classification performance. The Stratified *k*-fold cross-validation is an extension of this method defined by Eq (2). It keeps the original dataset’s class ratio constant across all *k* folds which ensures that any single class will not be over selected as our target variable is imbalanced.

$$\begin{array}{c} CV_{k}=\frac{1}{k}\sum_{n=1}^{k}MSE_{n} \end{array}$$ (2)

where

- *k* = Number of folds.
- *MSE* = Mean Squared Error.
- *CV*_*k*_ = Averaged cross validation estimate of k-fold.

The observations in the held-out fold are used to determine the mean squared error (MSE). *MSE*_1_, *MSE*_2_, …, *MSE*_*k*_ are the *k* estimations of the test error produced by the technique. These values are averaged to create the global *k*-fold CV estimate. In stratified k-fold, the percentage of samples from each target class in each fold must be similar to that of the entire set.

**Fig 2:**
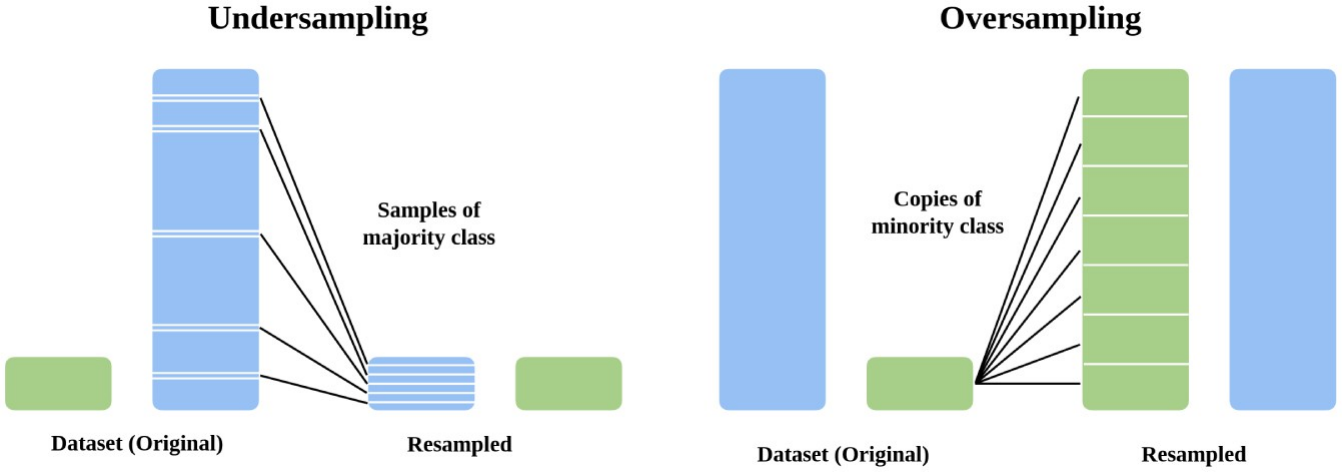
The process of Over-sampling and Under-sampling techniques.

Over-sampling techniques (Fig 2) replicate or add additional synthetic cases in the minority class randomly. In contrast, Under-sampling techniques (Fig 2) eliminate instances randomly in the majority class without any duplication. NearMiss is an Under-sampling technique which attempts to balance the distribution by removing the data point from the biggest class when two points in the distribution that belong to separate classes are very close to one another. While SMOTE is an Over-sampling technique which calculates the nearest neighbors for each minority class instances and randomly pick one and then creates a new instance.

**Fig 3:**
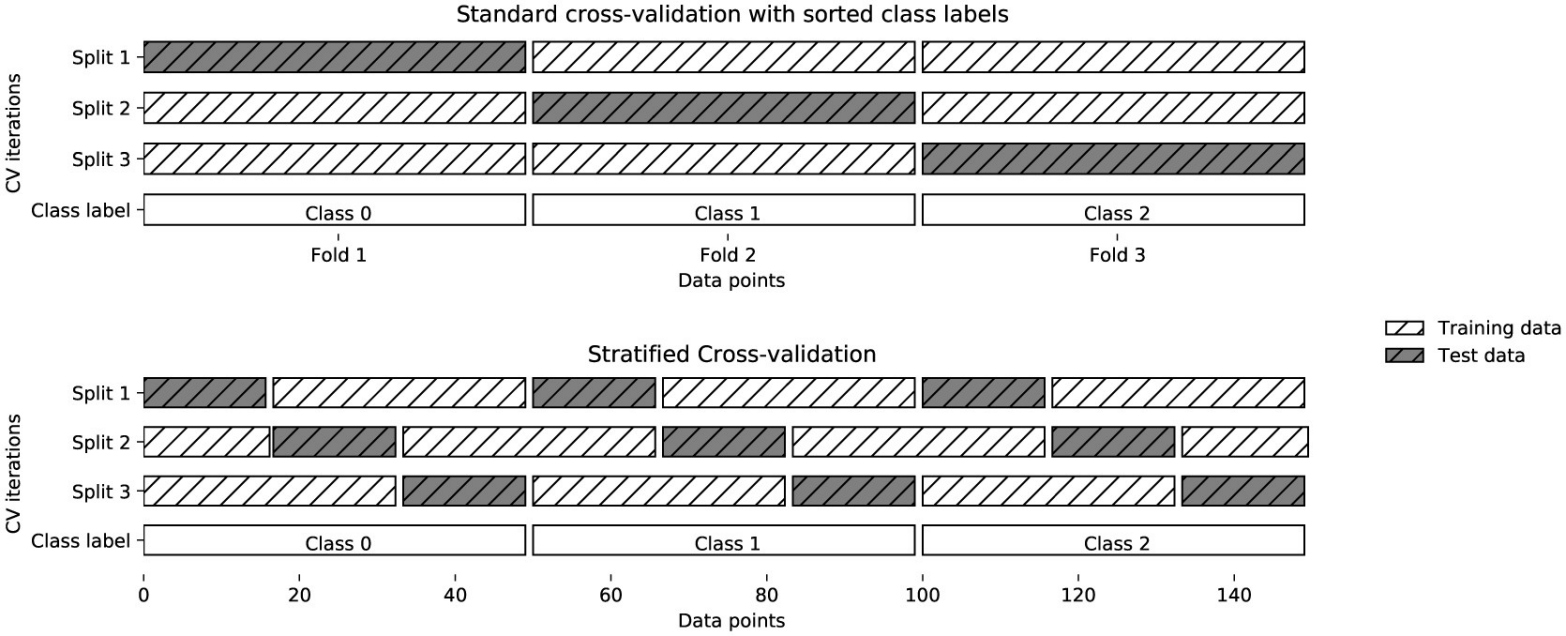
The step by step process of stratified approach with cross validation.

Stratified sampling divides the data sample classes with the same ratio of training and test subsets (Fig 3).

#### Classification algorithms

To automatically classify an exam, several machine learning classifiers were studied. More specifically, Support Vector Classifier from Support Vector Machine (SVM) [31, 32], K-Nearest Neighbors (KNN) [33], Random Forest (RF) [34, 35], Extremely Randomised Tree (ERT) [34, 36], Gradient Boosting (GB) [34, 37], Decision Tree (DT) [38], Multi-Layer Perceptron (MLP) [39], eXtreme Gradient Boost (XGB) [40], Light Gradient Boost Machine (LGBM) [41] and Stacked generalization (Stacking) [10, 34] have been used. As can be noticed, well-known traditional machine learning classifiers, ensemble methods such as boosting and Stacking, and the basic MLP neural network architecture are considered. These machine learning classifiers are fitted during the training phase. The classification accuracy (ACC) was considered to evaluate the different approaches. The hyper-parameters considered in this study are summarized in Table 2.

**Table 2:** Hyper-parameters used in different classifiers.

Techniques	Hyper-parameters
SVM	kernel = ‘rbf (radial basis function)’, degree = 3, gamma = ‘scale’, decision_function_shape = ‘ovr (one-vs-rest)’
KNN	n_neighbors = 5, weights = ‘uniform’, leaf_size = 30, p = 2, metric = ‘minkowski (euclidean_distance)’
RF	n_estimators = 100, criterion = ‘gini’, max_features = ‘sqrt’
ERT	n_estimators = 100, criterion = ‘gini’, max_features = ‘sqrt’
GB	loss = ‘log_loss’, learning_rate = 0.1, n_estimators = 100
DT	criterion = ‘gini’, splitter = ‘best’
MLP	hidden_layer_sizes = (100,), activation = ‘relu’, solver = ‘adam’, alpha = 0.0001, learning_rate = ‘constant’, learning_rate_init = 0.001, power_t = 0.5, max_iter = 200
XGB	base_score = 0.5, gamma = 0, learning_rate = 0.1, max_depth = 10, n_estimators = 100, objective = ‘binary:logistic’
LGBM	boosting_type = ‘gbdt’, num_leaves = 31, learning_rate = 0.1, n_estimators = 100
Stacking	estimators = selected_best_from_experiments, final_estimator = XGB, cv = 5

In addition, Level 1 meta learners for Stacked generalization model are determined based on the accuracy of individual classifiers. If the validation accuracy of a classifier is more than 80% then that classifier is stored for using it in the first level of stacked model. eXtreme Gradient Boost classifier is used as the final predictor in Level 2.

Once the training phase is completed, the saved models are tested with new data, in which the classification model and imbalance techniques are also passed to determine which combination provides the best results. After that, the important features which are more convenient have also been checked.

#### Evaluation

Below are listed the different metrics used to evaluate the performance of a classifier. The experimental assessment has been performed on a MacBook Pro (Retina, 15-inch, Mid 2015). The configuration includes a 2.5 GHz Quad-Core Intel Core i7 processor, 16 GB 1600 MHz DDR3 memory and Intel Iris Pro 1536 MB graphics.

#### Boxplots

Boxplots are useful to determine the spread of data, to identify outliers and to compare the distributions. In fact, Boxplots work best when it is necessary to compare the distributions of different groups. Boxplots summarize the facts concisely, and the locations of the box and whisker markings make it simple to compare different groups [42].

#### Confusion matrix

**Fig 4:**
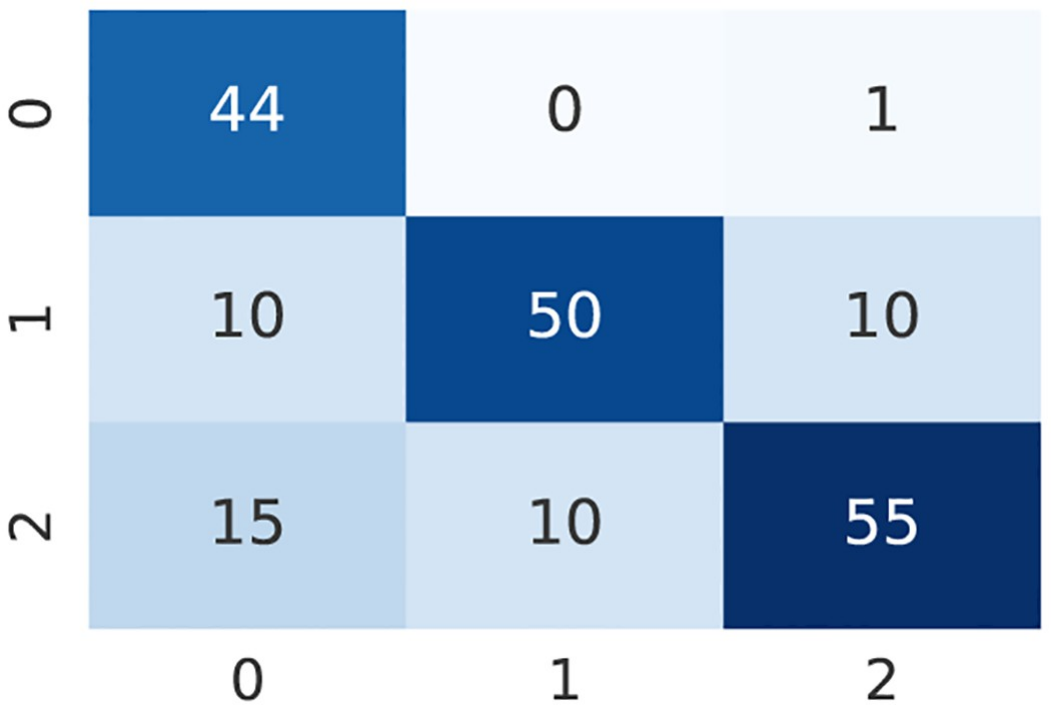
An example of confusion matrix. 0 denotes patients with normal DE-MRI, 1 patients with Myocardial Infarction, and 2 patients with Myocarditis.

A confusion matrix is a table that describes how well a classification model, or classifier, performed on a set of test data for which the true values are known. Although the confusion matrix is straightforward to comprehend, the terminology used to describe it can be perplexing [43]. An example of a confusion matrix for our study is depicted in Fig 4, where 0 denotes patients with normal DE-MRI (and then other clinical condition), 1 patients with Myocardial Infarction, and 2 patients with Myocarditis.

#### Accuracy

Accuracy (ACC) is the ratio of correct predictions that can be displayed as a percentage. It measures how accurately a model can predict on the entire set of data. From mathematical point of view, it is defined as follows:

$$\begin{array}{c} ACC=\frac{TP+TN}{TP+FP+TN+FN} \end{array}$$ (3)

where *TP* means True Positive, *TN* True Negative, *FP* False Positive, and *FN* False Negative. This value can be computed directly from the confusion matrix. Indeed the numerator is the sum of all the True elements which are on the main diagonal of the confusion matrix, while the denominator is the sum of the entries of the matrix. For the confusion matrix shown in Fig 4, the obtained accuracy is thus $ACC=\frac{149}{195}\approx 76.41\%$.

#### Precision

Precision measured the correctly predicted positive outcomes from the model. In other point of view, it demonstrates how many accurate and positive predictions there were. From a mathematical point of view, it is defined as follows:

$$\begin{array}{c} Precision=\frac{TP}{TP+FP} \end{array}$$ (4)

#### Recall

Recall, called also sensitivity, is a measurement of the proportion of positive instances that the classifier correctly predicted out of all the positive cases in the data. From a mathematical point of view, it is defined as follows:

$$\begin{array}{c} Recall=\frac{TP}{TP+FN} \end{array}$$ (5)

#### F1-Score

A measurement that combines recall and precision is called the F1-Score. It is often defined as the harmonic means of precision and recall. From mathematical point of view, it is defined as follows:

$$\begin{array}{c} F1-Score=2\times \frac{Precision\times Recall}{Precision+Recall} \end{array}$$ (6)

## Results

The average accuracies of all combinations of machine learning and data unbalancing techniques are summarized in Table 3. It can be noticed that the combination of Over-sampling and Stacking (an accuracy above 97%), as well as Light Gradient Boosting Machine (an accuracy above 96%) are the methods providing the best results. The combinations of SMOTE with Stacking or LightGBM are both ranked just after.

**Table 3:** Accuracy comparison obtained with all combinations. Best result in bold. (Stratified denoted as STRF).

Techniques	Stratified (STRF)	Under-sampling + STRF	Over-sampling + STRF	NearMiss + STRF	SMOTE + STRF
SVM	82.2	70.6	84.17	76.2	82.87
KNN	76.2	70.7	81.75	69.3	84.92
RF	86.8	83.9	95.90	82.6	92.36
ERT	83.1	82.6	94.97	78.7	91.99
GB	86.1	79.3	96.09	79.3	91.99
DT	79.8	73.4	92.92	66.6	84.73
MLP	82.8	77.9	88.45	78	86.59
XGB	87.7	78.1	95.90	80.7	93.11
LGBM	84.9	83.2	96.1	78.1	93.3
Stacking	85.8	76	**97.96**	75.8	92.74

Focusing on Over-sampling+STRF (Stratified denoted as STRF), it can be observed that many machine learning algorithms provide a high level of accuracy. Indeed, Gradient Boosting (GB) has more than 96% of accuracy, Random Forest (RF) and eXtreme Gradient Boosting (XGB) have a classification accuracy above 95%, while Extremely Randomized Tree (ERT) and Decision Tree (DT) are slightly less efficient with an accuracy of 94.97% and 92.92%, respectively. Over-sampling is clearly the best imbalance data processing technique, as it outperforms all other techniques. It gives the best classification accuracy for nearly all machine learning algorithms, the only exception being the K-Nearest Neighbors (KNN) for which the best imbalance technique is SMOTE+STRF.

Table 4 shows the comparison of the execution time required to train (304 cases) and validate (25 cases) the different machine learning algorithms. We can notice that Stacking techniques need much more time than other techniques. However, for each new individual test case the trained model takes less than one second to classify the patients after getting the clinical information as input. In details, the model takes very few resources and time to perform a classification because clinical information is tabular data which requires less resources (in terms of hardware and execution time) than image processing (such as MRI). Thus, it is not necessary to have high-performance and therefore expensive hardware to run the process on new individual cases.

**Table 4:** Execution time of all combinations for training (304 cases) and validation (25 cases)—in seconds. (Stratified denoted as STRF).

Techniques	Stratified (STRF)	Under-sampling + STRF	Over-sampling + STRF	NearMiss + STRF	SMOTE + STRF
SVM	0.07	0.03	0.11	0.03	0.11
KNN	0.07	0.04	0.08	0.04	0.07
RF	1.67	1.62	1.87	1.72	1.91
ERT	1.23	1.25	1.38	1.23	1.50
GB	2.88	2.41	3.64	2.45	4.21
DT	0.04	0.05	0.02	0.02	0.03
MLP	2.76	1.50	4.47	1.56	4.62
XGB	2.90	1.33	3.32	1.47	2.49
LGBM	1.71	0.36	1.30	0.51	1.84
Stacking	129.29	33.88	154.19	33.13	163.20

**Fig 5:**
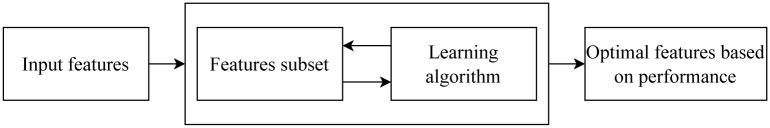
Wrapper method for feature importance identification.

In addition, the importance of each feature in the classification decision is obtained using a wrapper selection method. In this method, the decision-making process for choosing features depends on a particular machine learning algorithm that we are attempting to fit to a certain dataset (Fig 5). The five most important features are troponin, age, tobacco, sex, and FEVG as shown in Table 5 for Random Forest. This ranking is valid for all the classifiers. It is noteworthy that the two most decisive features, namely troponin and age, have an importance far beyond the others.

**Table 5:** Feature importance for the classification (using Random Forest).

Features Name	Importance (%)
Troponin	31.50
Age	30.8
Tobacco	12.90
Sex	8.43
NT-proBNP	7.28
FEVG	5.20
Familial disease	1.41
Overweight	1.15
Diabetes	0.66

**Fig 6:**
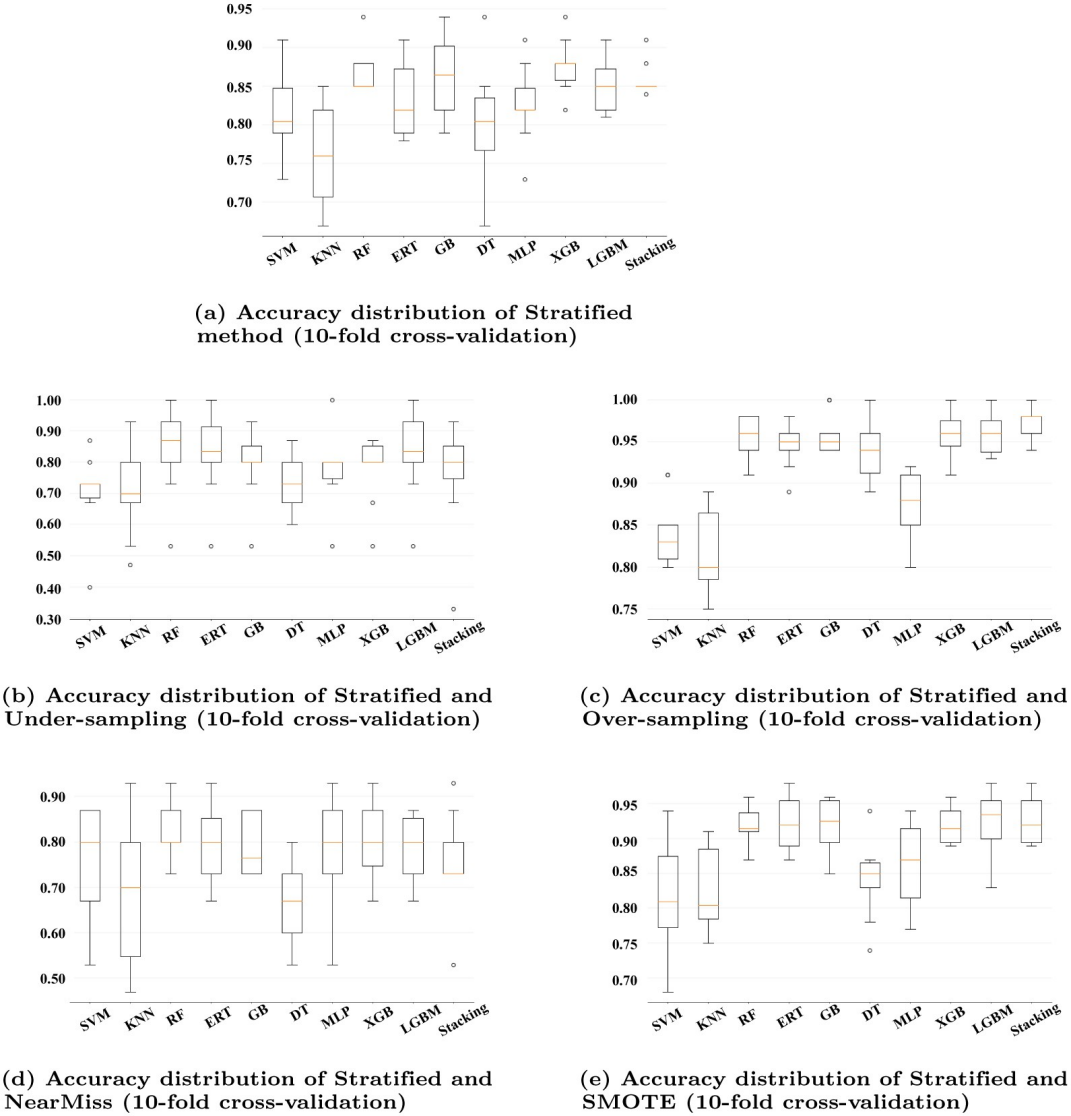
Comparison of accuracy of Support Vector Machine (SVM) classifier, K-Nearest Neighbors (KNN), Random Forest (RF), Extremely Randomised Tree (ERT), Gradient Boosting (GB), Decision Tree (DT), Multi-Layer Perceptron (MLP), eXtreme Gradient Boost (XGB), Light Gradient Boost Machine (LGBM) and Stacked generalization (Stacking). (a) Accuracy distribution of Stratified method (10-fold cross-validation), (b) Accuracy distribution of Stratified and Under-sampling (10-fold cross-validation), (c) Accuracy distribution of Stratified and Over-sampling (10-fold cross-validation), (d) Accuracy distribution of Stratified and NearMiss (10-fold cross-validation, (e) Accuracy distribution of Stratified and SMOTE (10-fold cross-validation).

Fig 6(a)–6(e) depict the accuracy distribution for the 10-fold cross-validation obtained for the different combinations of machine learning classifiers without and with data imbalance techniques. We can see that the accuracy distribution of LGBM with Over-sampling is better than SMOTE, with a less skewed distribution, while XGB has a symmetric distribution with an accuracy of 95.90% and an execution time of approximately 3.32 seconds. Overall, LGBM with Over-sampling provides a good distribution, good accuracy, and reduced execution time. On the other hand, Stacking with Over-sampling also provides an accuracy level superior to 97% and a lower execution time than SMOTE. However, the distribution with SMOTE is positively skewed.

From the accuracy comparison it was found that Stacking and LGBM performed better along with the combination of Over-sampling and SMOTE accordingly. Thus, it is interesting to check the confusion matrices of those combinations for more insights.

**Fig 7:**
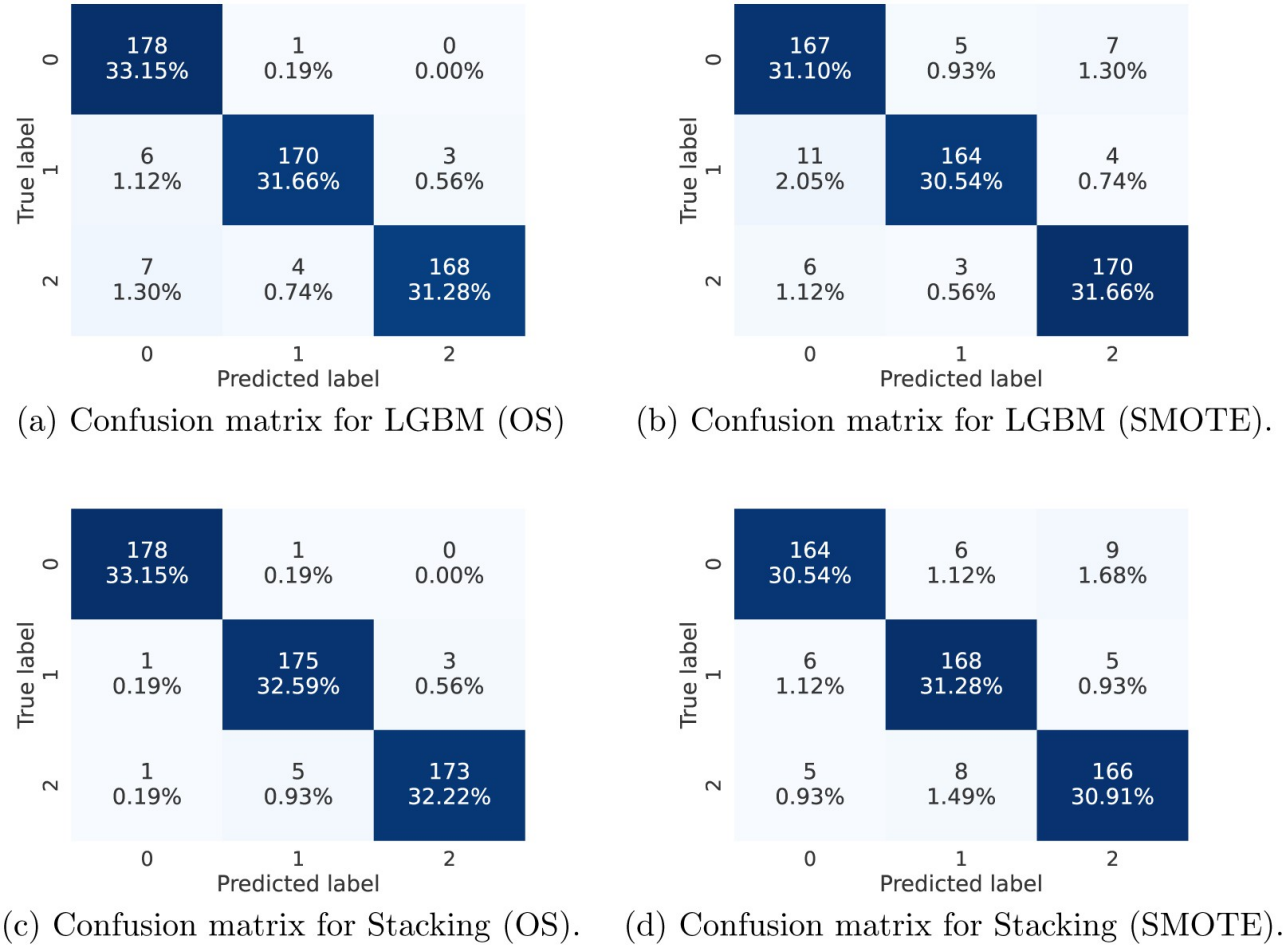
Comparison of the confusion matrices for the different approaches. 0 denotes patients with normal DE-MRI, 1 patients with Myocardial Infarction, and 2 patients with Myocarditis. (a) Confusion matrix for LGBM (OS). (b) Confusion matrix for LGBM (SMOTE). (c) Confusion matrix for Stacking (OS). (d) Confusion matrix for Stacking (SMOTE).

Fig 7(a)–7(d) show the confusion matrices from LGBM and Stacking along with the Over-sampling (OS) and SMOTE techniques, respectively. The number and the percentage of correctly classified patients are shown in each confusion matrix. It can be noticed that the values outside the diagonals, which represent the percentages of misclassified outcomes, are very small whatever the technique. Furthermore, ensemble methods performed better. More specifically, as shown in Fig 7(c), Stacked Generalization with Over-sampling (OS) provided an average classification accuracy above 97% (the sum of the diagonal values is 33.15 + 32.59 + 32.22 = 97.96%).

During the experiments, a 10 fold cross-validation technique was applied. Thus the sum of all folds is calculated in the confusion matrix. It can be noticed that the train:test dataset split ratio was 90:10 since a 10-fold cross-validation was used. In the case of the Over-sampling technique, it makes all the classes with the same number of data samples which is 179 samples (the size of the myocarditis class). Thus, 10% of the 179 cases of a class were used each time for testing, which means that 17 or 18 samples from each class are counted as test samples in a fold. In fact 9 folds will have 18 samples and one 17 samples. Overall, in the confusion matrix each class has 179 samples and represents 33.33% of the test samples. Therefore, the nearer a diagonal percentage to this value the better the classification. It can be observed from Fig 7(c) that the class of patients with normal DE-MRI has the highest average accuracy with 33.15%, while myocardial infarction and myocarditis have percentage values of 32.59% and 32.22%, respectively. Thus, for each class, there are very few misclassified cases.

For a finer analysis of the classification performance we also calculated the Precision, Recall and F1-score values for each class and combination of machine learning classifiers and data imbalance technique. Table 6 summarizes the values obtained using macro-averaging, as we deal with a multi-class classification problem. The mean and standard deviation over all classes for a given combination were provided. It is clear that Over-sampling outperforms SMOTE for a given classifier, almost always providing better results regardless of the metric. Indeed, the only case where SMOTE is better is for the recall for the class 2 (myocarditis). Comparing the classifiers, we can see that with Over-sampling, Stacking is better than LGBM, whereas when using SMOTE the classifier LGBM gives a better mean value but a larger standard deviation. Another point to note is that the Stacking classifier provides more stable prediction for the different classes, with the lowest standard deviations for precision and recall.

**Table 6:** Metrics calculated from the confusion matrices.

Precision	LGBM-OS	LGBM-SMOTE	Stacking-OS	Stacking-SMOTE
Class 0	93.19	90.76	98.89	93.71
Class 1	97.14	95.35	96.69	92.31
Class 2	98.25	93.92	98.30	92.22
Mean	96.19	93.34	97.96	92.75
Std dev.	2.66	2.35	1.14	0.84
Recall	LGBM-OS	LGBM-SMOTE	Stacking-OS	Stacking-SMOTE
Class 0	99.44	93.30	99.44	91.62
Class 1	94.97	91.62	97.77	93.85
Class 2	93.85	94.97	96.65	92.74
Mean	96.09	93.30	97.95	92.74
Std dev.	2.96	1.68	1.40	1.12
F1-Score	LGBM-OS	LGBM-SMOTE	Stacking-OS	Stacking-SMOTE
Class 0	96.22	92.01	99.16	92.66
Class 1	96.05	93.45	97.22	93.07
Class 2	96.00	94.44	97.46	94.48
Mean	96.09	93.30	97.95	92.74
Std dev.	0.12	1.22	1.06	0.30

## Discussion

In this study, automatic classification of patients after suspicion of myocardial infarction or myocarditis has been performed and assessed with multiple classifiers. In addition, overfitting and data unbalancing issues were also considered during this work. Therefore data imbalance techniques such as Under-sampling, Over-sampling, NearMiss and SMOTE were evaluated with the combination of machine learning classifiers. In addition, 10-fold cross-validation was performed to ensure that no overfitting may occur during training. It was found that LGBM and Stacking, both with Over-sampling, yielded the best accuracies for the classifications of the patients on our dataset. Whatever the technique of classification, Over-sampling improves the accuracy, as does the SMOTE approach. A drawback of Stacking approach is the processing time, so the LGBM approach with Over-sampling can be an excellent alternative. According to the confusion matrices, the advantage of Stacking compared to SMOTE is the lower number of labels 1 (MI) or 2 (myocarditis) classified as 0 (normal DE-MRI exam).

Each model provides as output a probability for each class, and one kept the highest probability for the predicted patient condition among myocardial infarction, myocarditis and other condition. It was observed that misclassification of health status occurred mainly when a patient had a MI or a myocarditis, whereas cases with normal DE-MRI were classified correctly. Then, we assume that the number of samples as well as the large-scale variant of the model for both diseases can help minimize the misclassification rate.

The evaluation of different machine learning algorithms was provided, including the processing of the data imbalance that actually exists in clinical practice. Then an automatic diagnostic tool was provided which allows the pathological classification that could help the doctors to distinguish quickly (and before doing specific imaging modality such as MRI) between myocarditis and myocardial infarction. Among the considered clinical data, troponin and age are the most important features, whereas sex, NT-proBNP, and LVEF from echocardiography also have a significant influence in the classification. It is not a surprise to discover troponin or tobacco, but we can notice that NT-proBNP and FEVG calculated from echocardiography is not selective for these 3 classes. Indeed concerning FEVG, one can imagine that this value is lower for patient with MI. As a future work, based on the same concept, other cardiovascular diseases managed in a emergency department, such as tako-tsubo syndrome or cardiac tamponade, can be included in this tool. Another possible update of our tool is to provide a score of confidence in the results.

## Conclusion

Patients attending an emergency department with severe chest pain and suspicion of myocardial infarction or myocarditis are usually referred to medical imaging, and more particularly cardiac MRI, to refine the diagnosis. However, one of the barriers providing immediate care is the delay between admission of patients to the emergency department and the actual MRI scan. To minimize the delay, it could be interesting to have a pre-classification of the diseases without waiting for the imaging examination, and then starting the specific medical care. Thus, our study provides a clear approach of solving that issue by classifying the patients using only clinical data. This classification must be considered as additional information provided to the clinician, and must not replace other features, in particular sanguine samples. The proposed approach considers the practical situation of data imbalance and suggests to incorporate the Stratified techniques. Thus, it minimizes the data bias during the training of the model. In fact, we found that learning from the results of meta-learners which is known as Stacked Generalization provides higher performance and minimizes the error bias. We also found that the Over-sampling approach provides the best performance and thus we can conclude that more data samples may improve the accuracy. However, Over-sampling may cause overfitting. Without Over-sampling, eXtreme Gradient Boosting (XGB) with Stratified k-fold Cross-Validation provides the highest accuracy (87.7%), followed by Random Forest with 86.8% of accuracy. It can be concluded that the classification of patients with myocardial infarction or myocarditis based on solely clinical data is possible, and more data samples should allow us to have more credibility in the results. Then as future works, other diseases managed in emergency department can be considered, such as the takotsubo syndrome or cardiac tamponade. Moreover, a limitation of our work is that the data arose from only one clinical structure, and a multi-centric study should be considered in order to evaluate the robustness of our approach. Finally, the worst result is to wrongly classify a patient having an hyperenhancement on DE-MRI, and then efforts must be done to discard that.
